# Healthy indoor air is our fundamental need: the time to act is now

**DOI:** 10.5694/mja2.51768

**Published:** 2022-11-13

**Authors:** Lidia Morawska, Guy B Marks, Jason Monty

**Affiliations:** ^1^ Queensland University of Technology Brisbane QLD; ^2^ Woolcock Institute of Medical Research Sydney NSW; ^3^ Liverpool Hospital Sydney NSW; ^4^ University of Melbourne Melbourne VIC

**Keywords:** Disease transmission, infectious, Air pollutants

Inadequate management of indoor air quality may not be obvious, but the disastrous consequences certainly are

According to estimates by the World Health Organization, polluted outdoor air kills over 7 million people annually.^1^ In 2021, the WHO published new air quality guidelines to serve as the basis for setting or updating national ambient air quality standards.^2^ This document can also be the basis for setting national indoor air standards, as the recommended air pollution levels apply both to outdoor and indoor air. Will national jurisdictions update their indoor air quality (IAQ) standards? The shocking reality is that most countries, including Australia, do not have any IAQ standards or even plans to establish them. The handful of countries that have standards do not have the means and procedures to enforce them; therefore, they do not serve their purpose.^3^

## What are the consequences of poor indoor air quality?

The burden of disease due to indoor air pollution in terms of disability‐adjusted life‐years in 26 European countries was demonstrated in the IAIAQ project.^4^ Numerous studies have quantified the negative impact of poor air quality in buildings on health, general wellbeing, and productivity.^5^ In Australia, the pre‐pandemic costs attributable to respiratory, neurological and other symptoms and illnesses arising from exposure to hazardous gases and particles (both biological and non‐biological) in the indoor environment were certainly above the $12 billion per year calculated in a 2001 study.^6^

In addition to pollutants from indoor and outdoor anthropogenic sources, other types of pollutants are those that humans emit. We continuously exhale carbon dioxide (CO_2_) and generate particles during all our respiratory activities, at a rate and size dependent on the activity.^7^ If pathogens (viruses or bacteria) are present in the respiratory tract, they are emitted as a component of the particles. The predominantly small size of these particles (most of them are < 1 μm) means that they can float in the air for prolonged periods and travel substantial distances within an indoor environment; if a susceptible person inhales these pathogen‐laden particles, they can become infected. This process is called airborne transmission of respiratory infections, which the coronavirus disease 2019 (COVID‐19) pandemic brought dramatically to our attention.^8^ Airborne transmission is considered the dominant mode of transmission of numerous respiratory infections.^9^ Of course, this is not a new risk, it has been with us forever, but was not considered, not recognised, and ignored. Globally, before the COVID‐19 pandemic, acute respiratory illnesses such as colds and influenza accounted for an annual estimated 300 million lower respiratory infections, resulting in more than 2.7 million deaths and economic losses of billions of dollars.^10^ Similar to other countries, viral respiratory infections are a major cause of morbidity and mortality in Australia.^11^

The economic cost of these infections is high; non‐influenza respiratory infections cost global communities tens of billions of dollars annually. The estimated cost of acute lower respiratory infections in the European Union totalled €46 billion in 2011;^12^ the economic burden from all lower respiratory infections in Australia exceeded $1.6 billion in 2018–19.^13^ Although it is unlikely that we could eliminate respiratory infections by controlling airborne transmission in shared indoor spaces, we can substantially reduce them. If hospital admissions occasioned by these diseases could be halved by limiting airborne infections, tens of thousands of Australians would remain healthy, saving hundreds of millions of dollars each year.

Times of crisis expose the limitations of internal atmospheres. Along the Australian south‐eastern seaboard in 2019–2020, buildings failed to protect people from bushfire smoke.^14^ In the COVID‐19 pandemic, countless congregational settings (offices, schools, factories, residential aged care facilities, cruise ships etc), where most of the population spends a substantial fraction of the day working, studying, travelling, enjoying entertainment, resting or undergoing medical care as part of their daily lives, allow virus‐laden particles to spread through indoor air.^15^ Inadequate management of internal atmospheres might not be obvious, but the disastrous consequences certainly are.

## Why is indoor air quality so neglected?

Why is clean indoor air not considered of utmost importance to our health and wellbeing? After all, we spend more than 90% of our lives in buildings, breathing indoor air about 12 times a minute. The simplest answer is because IAQ is a regulatory “no man's land”.

Globally, IAQ presents a complex political, social and legislative challenge, with lack of an open, systematic and harmonised approach. Even though the Australian Standard AS1668 and the National Construction Code specify ventilation system requirements, these are for new buildings and consider only outside air provision; they are not consistent with the WHO guidelines, do not consider air quality, and are not enforced. In Australia, as in most countries, there is no single national government authority with responsibility for IAQ, and any relevant legislation is at the discretion of individual states and territories, not the Commonwealth. In individual states and territories there are no bodies directly responsible for IAQ; responsibilities are spread between different organisations. For example, the Department of Education is responsible for IAQ in schools, and the Department of Health governs IAQ in health care facilities. It is a similar story for hospitality venues, office buildings, and retail. Further, occupational and residential environments are treated differently, and information on assessment of the indoor environment is often available only to building owners and treated as confidential.

Importantly, there are no performance standards for indoor air, only design and operation standards. Although outdoor air legislation is based on performance standards (through compliance with concentration levels of pollutants prescribed by the standards), indoor environment legislation is limited to design standards. Factors in the design include air exchange rate, filter specifications, and size of windows. Each factor is related to IAQ but is not the only one responsible for it; therefore, without actually measuring the performance of the building system, IAQ is unknown. This is not a new situation, the issue was raised more than 20 years ago,^16^ and despite the efforts of many air quality and public health experts, the situation has not changed.

## What should be done?

How can we secure good IAQ for Australians and minimise the risk of airborne infection transmission in shared indoor spaces? The simple answer is that we need enforceable IAQ standards (Box). Here, we will focus only on the aspects of the legislation relating to infection transmission — standards to protect against anthropogenic pollutants in indoor air are outside the scope of this article.

Box 1 **Recommendations to secure good indoor air quality (IAQ)**

**Table:** 

Establish a consistent national regulatory infrastructure for clean indoor air through the Federal Cabinet working with the states and territories through the National Cabinet. Establish an interdisciplinary panel of experts, including scientists, engineers, architects, and medical and public health professionals tasked with developing a foundation for IAQ standards that can be legislated and enforced. There are health‐based World Health Organization Air Quality Guidelines (WHO AQG) that apply to both outdoor and indoor air. However, since we cannot routinely measure the pollutants included in the guidelines in every shared indoor environment, we need to identify a set of pollutants that can be measured. Pollutants originating from human expiration indoors, including pathogens, lead to airborne infection transmission. They are not included in the WHO AQG and it is not feasible to routinely measure pathogens in these environments. Therefore, we need to identify proxy parameters for such pollutants that can be measured. Legislate the IAQ standards. Mandate that all new buildings are designed to meet these standards. Include protection against indoor air hazards with a particular focus on airborne infection control in the statements of purpose and definitions of all relevant Australian building design and building engineering standards, regulations and codes. Review and improve the existing building design and building engineering standards, regulations and codes to ensure that they enable compliance with the IAQ standards. Establish a national fund enabling the rollout of indoor environment modernisation measures addressing both immediate emergencies as well as a long term transition process towards all shared interiors meeting IAQ standards.

To put it simply, we need to remove our respiratory effluents (CO_2_ and respiratory particles) at a sufficiently high rate in relation to their production, so they do not accumulate in indoor air. A simple term for this action is “ventilation”. This is of course not a new concept: over 150 years ago, British nurse Florence Nightingale highlighted the need for ventilation “to keep the air he breathes [hospital patient] as pure as the external air without chilling him”.^17^ Even though the concept is simple, its implementation poses many challenges, as highlighted above in relation to indoor air standards in general. An added complexity is that there is no clear answer to the question of which parameter or pollutant should be selected as the basis for a standard targeting airborne infection transmission, and what the numerical value should be, because it is not feasible to directly monitor infectious pathogens in real time. Because ventilation plays an important role in controlling airborne infection transmission, a quantitative measure of ventilation is a key parameter to consider. Of the two aspects of ventilation presented in a 2020 article,^18^ sufficient ventilation (meaning enough ventilation) and effective ventilation (meaning ventilation reaching everywhere and air flow not passing from person to person), the former is the best contender for a standard. The WHO has already recommended a minimum ventilation rate for non‐residential settings of 10 L/s per person.^19^ This is a good starting point to consider for an indoor air standard. Importantly, however, for the standard to be enforced, ventilation needs to be measured in all shared spaces. Although technologies for this are already in place in most modern mechanically ventilated buildings (relying on mechanical systems to bring in air from outside), ventilation rates are not considered in relation to health, and often not in relation to the number of occupants or their activities. One way to assess the quality of ventilation is by a visual display of CO_2_ concentration: if it increases above an accepted threshold level in relation to the outdoor concentration, it means that ventilation is inadequate. In this way, CO_2_ readings are a proxy for ventilation, and like any proxy, it has limitations. However, CO_2_ sensors are now readily available, low cost and robust, and can be used in every interior in the same way as smoke alarms.

But do we know whether this is enough to adequately lower the risk of infection and whether it is effective for a particular pathogen or variant? To answer these questions, we assessed the individual infection risk of an exposed person and the event reproduction number — the expected number of new infections arising from a single infectious individual at the event — for a range of respiratory pathogens.^20^ We showed that for the most infectious pathogens, including adenovirus, untreated tuberculosis, and severe acute respiratory syndrome coronavirus 2 (SARS‐CoV‐2), even a high ventilation rate of 14 L/s^‐1^ p^‐1^ may be insufficient to maintain event reproduction numbers below one in a fully susceptible population, depending on indoor occupant activities (our calculations were done for a classroom and barracks). This means that in a setting with a high density of occupants and for highly infectious pathogens, ventilation alone cannot adequately reduce the risk of infection. Fortunately, there is a solution to supplement ventilation: disinfecting the air with germicidal ultraviolet (UV) radiation, which kills or deactivates pathogens.^21^ This old and well established technology was first successfully used in classrooms in the United States in the 1930s to lower the risk of airborne spread of measles^22^ — known as the most infectious disease until the Delta variant of SARS‐CoV‐2 surpassed it. This technology (UV‐C 254 nm) does not generate new pollutants in the air; is silent, robust (low maintenance) and low cost; has low energy requirements; and is already covered by international and Australian standards (AS/NZS IEC 62471:2011) as well as workplace safety standards.^23^ Its extension to far UV radiation (222 nm), which does not penetrate the skin, opens greater opportunities for use, and if utilised in shared spaces, it could be doing to air what is done to water — every drop of water we drink is disinfected. The technology is there, it is proven, but we need a social licence to use it.

In our recent article, the paradigm change of introducing IAQ regulations aimed at airborne infection transmission and modernising buildings to improve IAQ was compared with the transformation of sanitation infrastructure in the United Kingdom in the 19th century.^9^ It was not an easy task to convince authorities of the need for clean water and the role of contaminated water in infection transmission. Ultimately, the authorities and the community were convinced when, during the cholera outbreak in London in 1854, British doctor John Snow persuaded town officials to remove the handle of the local water pump. Locals could not drink that water and the outbreak was contained. This changed the approach to water sanitation in Britain and, ultimately, the whole world, with enormous demonstrable public health benefits and corresponding economic dividends through health care savings.^24^

## Is it feasible to provide clean, healthy air in buildings across Australia?

Estimates suggest that investment in new generation management systems to address airborne infections would likely result in less than a 1% increase in the construction cost of a typical building.^25^ In addition, the actual challenge and investment required to effect the modern reform would be much lower than the effort required in the United Kingdom to modernise the water system because Australia already has a sophisticated building infrastructure, public health regulatory frameworks, and public health law mechanisms to support the required advances. All buildings, public and private, will require modernisation, which will take time, but it is not a case of building from nothing. We must act now, starting with setting appropriate IAQ standards. Will the COVID‐19 pandemic, with its countless outbreaks in shared spaces lacking adequate infection control measures, be the “pump handle moment” in Australia in relation to airborne infection transmission?

## Open access

Open access publishing facilitated by Queensland University of Technology, as part of the Wiley ‐ Queensland University of Technology agreement via the Council of Australian University Librarians.

## Competing interests

No relevant disclosures.

## Provenance

Commissioned; externally peer reviewed.
